# Discrimination in an “equal country”—a survey amongst Swedish final-year medical students

**DOI:** 10.1186/s12909-022-03558-6

**Published:** 2022-06-27

**Authors:** Lotta Velin, Michelle S. Chew, Laura Pompermaier

**Affiliations:** 1 Centre for Teaching & Research in Disaster Medicine and Traumatology (KMC), Department of Biomedical and Clinical Sciences, Linköping University, Johannes Magnus väg 11, 583 30 Linköping, Sweden; 2 Division of Clinical Chemistry and Pharmacology, Department of Biomedical and Clinical Sciences, Faculty of Medicine and Health Sciences, Linköping University, Linköping, Sweden; 3 ANOPIVA US, Region Östergötland, Anaesthetics, Operations and Specialty Surgery Center, Linköping, Sweden; 4 Department of Hand Surgery, Plastic Surgery and Burns, Linköping University, Linköping, Sweden; 5 Department of Biomedical and Clinical Sciences, Linköping University, Linköping, Sweden

**Keywords:** Discrimination, Medical student, Survey, Sex, Gender, Ethnicity

## Abstract

**Background:**

Discrimination due to gender and ethnicity has been found to be widespread in medicine and healthcare. Swedish and European legislation list seven discrimination grounds (age, sex, ethnicity, religion, sexuality, non-binary gender identity, and disability) which may intersect with each other; yet these have only been sparsely researched. The aim of this study was to assess the extent of discrimination, based on these seven discrimination grounds, amongst final-year medical students in Sweden.

**Methods:**

A web-based survey, based on the CHERRIES-checklist, was disseminated to course coordinators and program directors in charge of final year medical students at all seven medical schools in Sweden. Quantitative data were analyzed using descriptive statistics, Fisher’s exact test, and logistic regression. Free-text answers were analyzed thematically using the “Master Suppression techniques” conceptual framework.

**Results:**

Of the 1298 medical students contacted, 247 (19%) took part in the survey. Almost half (*n* = 103, 42%) had experienced some form of discrimination, and this difference was statistically significant by gender (*p* = 0.012), self-perceived ethnicity (*p* < 0.001), country of birth other than Scandinavia (*p* < 0.001) and visible religious signs (*p* = 0.037). The most common type of discrimination was gender-based (in 83% of students who had experienced discrimination), followed by age (48%), and ethnicity (42%). In the logistic regression, women/non-binary gender (*p* = 0.001, OR 2.44 [95% CI 1.41–4.22]), country of birth not in Scandinavia (*p* < 0.001, OR 8.05 [2.69–24.03]), non-Caucasian ethnicity (*p* = 0.04, OR 2.70 [1.39–5.27]), and disability (*p* = 0.02, OR 13.8 [1.58–12040]) were independently associated with discrimination. Half of those who had experienced religion-based discrimination and nearly one-third of victims of ethnicity-based discrimination reported “large” or “extreme” impact of this. Clinical staff or supervisors were the most common offenders (34%), closely followed by patients and their relatives (30%), with non-Caucasian respondents significantly more likely to experience discrimination by patients (*p* < 0.001).

**Conclusions:**

Discrimination appears to be frequent in medical school, even in one of the world’s “most equal countries”. Discrimination is most commonly gender- or ethnicity-based, with ethnicity- and religion-based discrimination appearing to have the largest impact. Future research should continue to evaluate discrimination from an intersectional perspective, adapted for local contexts and legislations.

**Supplementary Information:**

The online version contains supplementary material available at 10.1186/s12909-022-03558-6.

## Introduction

Discrimination in medicine is widespread and can manifest itself in various forms, both among patients and health workers, such as inequalities in access to healthcare services among people of disadvantaged socio-economic backgrounds, or micro-aggressions toward healthcare workers of underrepresented genders or ethnicities [1, 2]. A systematic review including international studies, showed that 59.4% of medical students had experienced at least one form of discrimination or harassment during training [3]. Previous studies among medical students have mainly investigated sex-based discrimination [3, 4]. Regardless of its cause, discrimination can lead to detrimental mental health effects [5], affect performance, and influence the choice of career paths [6, 7].

On the Global Gender Gap Index 2021 [8], Sweden ranks as the fifth most gender-equal country in the world. Women make up approximately 50% of the country’s physicians, and 60% of medical graduates [9]. In Sweden, there is a long track record of institutional efforts to ensure gender parity; yet, as shown by over 10,000 testimonies from women physicians during the 2017 “MeToo” movement, gender-based discrimination and harassment is still widespread [10]. In accordance with non-discrimination laws mandated by the European Union (EU) [11], Swedish legislation lists seven discrimination grounds: age, sex, ethnicity, religion, sexuality, non-binary gender identity, and disability [12], although many of these remain sparsely investigated. The frequent notion of Sweden as a relatively “gender-equal” country, spurs questions regarding the extent to which discrimination, based on gender and other discrimination grounds, still exists, and how they manifest. The goal of this study was to quantify the extent of discrimination, based on these seven discrimination grounds, amongst final-year medical students in Sweden, and to qualitatively assess how it manifests through the Master Suppression techniques framework [13]. As a secondary goal we aimed to explore how students were affected by discrimination during medical school.

## Methods

### Survey instrument

The survey was designed and distributed in accordance with the CHERRIES protocol for online surveys [14]. The survey (Table 1, Additional file 1) was modified from an existing survey on gender-based discrimination used in India and Brazil. Contextual adaptations according to Swedish and European legislation, medical education structure, and health system structure were made, including expansion to all discrimination grounds The survey was translated and back-translated from English to Swedish by native Swedish- and English-speaking team members. Although the terms “sex” and “gender” are sometimes used interchangeably, “sex” refers to the biological characteristics (female/male), and “gender” to the social construct and associated cultural norms (woman/man/non-binary) [15]. In Swedish, no such clear division of concepts is commonly used; hence the survey only includes a question on self-identified gender, and the term “gender” was therefore used to describe the study results referring to man/woman/non-binary gender. For the logistic regression analysis, the gender variable was dichotomized to “men” and “non-men”, where “non-men” included women and non-binary respondents. The question “*do you think others perceive you as Swedish?*” dichotomized the variable “Ethnicity” as “Caucasian”/ “Non-Caucasian”. The survey was designed in REDCap [16] using a branching logic for adaptive questioning, ranging from 13 to 28 items depending on the responses. A completeness check was used. View rates and participation rates could not be calculated since IP addresses or personal contact information were not allowed to be collected due to ethical reasons. A preliminary outline of the survey was sent to an independent focus group, consisting of medical professionals at Linköping University Hospital with no research experience in the field of discrimination, for test feasibility and adequacy of the questions. The final form was outlined once majority consensus was reached among the coauthors, before the commencement of the study (i.e. April 26^th^ 2021).

### Study population and survey distribution

The target population was all final-year medical students (tenth and eleventh semester) in Sweden’s seven medical schools. The final year (consisting primarily of clinical courses) was selected as students in earlier stages of medical school were expected to have had less clinical exposure and therefore limited ability to complete the survey, and as previous literature indicates that medical students primarily appear to experience discrimination and harassment in clinical settings [3, 4].

Program directors and course coordinators at each of the seven medical schools were approached and provided standardized information about the study by email. They were requested to disseminate the study amongst students with a standardized recruitment strategy using an e-mail with an information, an invitation letter and a recruitment graphic (Additional file 1, Fig. 1). All program directors and course coordinators were sent at least one reminder and encouraged to send reminders to students also. Data regarding the number of students in each class were collected and used to calculate the target population.

### Data analysis

Data were analyzed using descriptive statistics (median, interquartile range, frequencies, and percentages), and Fisher’s exact test using Stata (StataCorp, 16.0) [17]. Logistic regression was used to assess the relationship between the dependent variable experience of discrimination (yes/no) and independent variables age (years), gender (women/men/non-binary gender), self-perceived ethnicity (Caucasian/non-Caucasian), country of birth (in Scandinavia/not in Scandinavia), visible signs of religious belief (yes/no), visual signs of disability (yes/no). Odds ratios (OR) and 95% confidence intervals (CI) are presented. Missing data were listwise deleted to conduct the logistic regression. Incomplete answers or illogical answers (where one answer contradicted another) were removed. Free-text responses were analyzed using a simplified thematic analysis, where all responses were reviewed, meaning-bearing units extracted and sorted under themes based on the conceptual framework of “Master Suppression techniques”, developed by Nissen and Ås, to classify discrimination techniques (Table 2, Additional file 1) [13], and citations were used to exemplify the themes. Brief demographic descriptions of the quoted participants were included but limited to a minimum level to avoid risk of personal identification.

### Ethical approval

All methods were performed in accordance with the Declaration of Helsinki. Participation was voluntary and anonymous. The Swedish Ethical Review Authority determined the study to be exempt from review, and that written, informed consent was not required, and could be assumed by the act of partaking in the survey (study number: 2021-01302). All data is presented at an aggregated level and individual respondents are unidentifiable.

## Results

Of the 14 course directors contacted, 13 agreed to distribute the survey amongst students. Of the 1298 medical students contacted, 247 (19.0%) responded to the survey; 55.1% (*n* = 136) of respondents were in the 10^th^ semester and 44.9% (*n* = 111) were in the 11^th^ semester. Most respondents were women (*n* = 147, 59.5%), born in Sweden (*n* = 204, 82.6%), self-perceived as Caucasian (*n* = 184, 74.5%), without visible religious signs (*n* = 214, 86.6%), heterosexual (*n* = 206, 83.4%), and without visible disability (*n* = 240, 97.2%) (Table 1). Median age was 26 (IQR 24–28).

**Table 1:** Demographic characteristics of study participants

	**N (%)**
**Gender**	
Women	147 (59.5)
Men	97 (39.3)
Non-binary gender / other /don’t want to state	3 (1.2)
**Country of birth**	
Sweden	204 (82.6)
In Scandinavia^a^, not Sweden	13 (5.3)
In Europe, not Scandinavia	14 (5.7)
Outside Europe	16 (6.5)
**Self-perceived ethnicity**	
Caucasian	184 (74.5)
Non-Caucasian	55 (22.3)
Not sure / don’t want to state	8 (3.2)
**Visible religious signs**	
No	214 (86.6)
Yes	23 (9.3)
Not sure	10 (4.1)
**Age** (median, IQR)	26 (24–28)
**Sexual orientation**	
Heterosexual	206 (83.4)
Bisexual	22 (8.9)
Homosexual	10 (4.0)
Other	9 (3.6)
**Presence of visible disability**	
No	240 (97.2)
Yes	7 (2.8)

^a^Scandinavia = Sweden, Norway, Denmark, Finland, Iceland, the Åland Islands and the Faroe Islands

### Extent of discrimination

Almost half (*n* = 103, 41.7%) had experienced some form of discrimination, with a statistically significant difference by gender (women: 49.0% vs men: 30.9%, *p* = 0.012), ethnicity (Caucasian: 34.2% vs non-Caucasian: 67.3%, *p* < 0.001), country of birth (outside Scandinavia: 36.4% vs in Scandinavia: 80.0%, *p* < 0.001), and visible religious signs (religious signs: 60.9% vs those without: 38.9%, (*p* = 0.037). There were no statistically significant differences by sexuality (heterosexual: 39.3% vs non-heterosexual: 53.7%, *p* = 0.216), and disability (disability: 85.7% vs no disability: 40.4%, *p* = 0.097) (Table 2).

**Table 2:** Characteristics amongst respondents who have experienced discrimination

	**N (%)**	***P*-value**
Women / Men	72 (49.0%) / 30 (30.9%)	0.012
Caucasian / non-Caucausian	63 (34.2%) / 37 (67.3%)	< 0.001
Born in Scandinavia^a^ / born outside Scandinavia	79 (36.4%) / 24 (80.0%)	< 0.001
Visible religious signs (yes/no)	14 (60.9%) / 83 (38.9%)	0.037
Presence of visible disability (yes/no)	6 (85.7%) / 97 (40.4%)	0.097
Heterosexual / non-heterosexual	81 (39.3%) / 22 (53.7%)	0.216

^a^Scandinavia = Sweden, Norway, Denmark, Finland, Iceland, the Åland Islands and the Faroe Islands

**Fig. 1:**
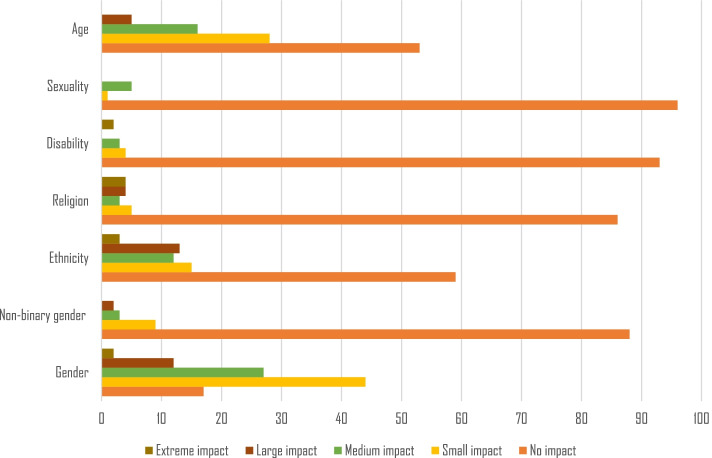
The self-reported impact of discrimination based on the seven discrimination grounds, amongst respondents who had experienced discrimination (*n* = 102*). *The sample size differs from the total sample (*n* = 103), since one respondent did not answer this question

The most common basis of discrimination was gender (*n* = 85, 82.5% of those who had experienced discrimination; 34.4% total prevalence), followed by age (*n* = 49, 47.5% of those who had experienced discrimination; 19.8% total prevalence), ethnicity (*n* = 43, 41.7% of those who had experienced discrimination; 17.4% total prevalence), religion (*n* = 16, 15.5% of those who had experienced discrimination; 6.5% total prevalence), non-binary gender identity or expression (*n* = 14, 13.6% of those who had experienced discrimination; 5.7% total prevalence), disability (*n* = 9, 8.7% of those who had experienced discrimination; 3.6% total prevalence), sexual orientation (*n* = 6, 5.8% of those who had experienced discrimination; 2.4% total prevalence) (Fig. 1). Of those born outside Scandinavia who had experienced discrimination, the most common discrimination ground experienced was ethnicity (*n* = 20/24, 83.3%), followed by gender (*n* = 17/24, 70.8%). Respondents that had experienced age-based discrimination were slightly older than those who had not, but this was not statistically significant.

In the logistic regression analysis, women/non-binary gender (*p* = 0.001, OR 2.44 [95% CI 1.41–4.22]), country of birth not in Scandinavia (*p* < 0.001, OR 8.05 [2.69–24.03]), non-Caucasian ethnicity (*p* = 0.04, OR 2.70 [1.39–5.27]), and disability (*p* = 0.02, OR 13.8 [1.58–12040]) were significantly and independently associated with discrimination (Table 3), while sexuality, age, and religion were not.

**Table 3:** Evaluation of factors associated with having experienced discrimination

	**Unadjusted Odds Ratio** **OR (95% CI, *p*-value)**	**Adjusted Odds Ratio** **OR (95% CI, *p*-value)**
**Age** (years)	1.00 (0.94–1.06, *p* = 0.95)	0.99 (0.93–1.06, *p* = 0.82)
**Sex / gender**		
Male	1	1
Females and non-binary respondents	1.84 (1.12–3.02, *p* = 0.02)	2.44 (1.41–4.22, *p* = 0.001)
**Self-perceived ethnicity**		
Caucasian	1	1
Non-Caucasian	3.27 (1.81–5.90, *p* < 0.001)	2.70 (1.39–5.27, *p* = 0.04)
**Country of birth**		
Scandinavia	1	1
Not Scandinavia	6.89 (2.70–17.57, *p* < 0.001)	8.05 (2.69–24.03, *p* < 0.001)
**Sexuality**		
Heterosexual	1	1
Non-Heterosexual	1.12 (0.83–1.50, *p* = 0.46)	1.20 (0.87–1.66, *p* = 0.26)
**Visible religious signs**		
No	1	1
Yes	1.47 (0.72–3.01, *p* = 0.28)	1.50 (0.66–3.39, *p* = 0.34)
**Visual signs of disability**		
No	1	1
Yes	8.72 (1.03–73.59, *p* = 0.04)	13.8 (1.58–120.40, *p* = 0.02)

Scandinavia = Sweden, Norway, Denmark, Finland, Iceland, the Åland Islands and the Faroe Islands

*OR* Odds Ratio, *CI* Confidence Interval

Gender-based discrimination most often had small (*n* = 44, 43.1%) or medium (*n* = 27, 26.5%) impact, although 13.7% (*n* = 14) reported large or extreme impact (Fig. 1). Sixteen (37.2%) of those who had experienced ethnicity-based discrimination reported large or extreme impacts, and of the 16 who had experienced religion-based discrimination, 8 (50.0%) reported large or extreme impact.

### Consequences of discrimination

The most common result of discrimination was reduced personal wellbeing (*n* = 81, 79.4%), followed by lowered self-confidence (*n* = 74, 72.5%), perceived limitation of future career options (*n* = 70, 68.6%), reduced academic performance (*n* = 65, 63.7%), and threatened personal safety (*n* = 39, 38.2%).

### Who discriminates?

The most common offenders of discrimination were medical staff/supervisors on clinical placements (*n* = 84, 33.7% of those having experienced discrimination), followed by patients/patients’ relatives (*n* = 74, 29.7% of those having experienced discrimination), and other medical students (*n* = 37, 14.9% of those having experienced discrimination). Respondents born outside of Scandinavia were more likely to report patients and their relatives as perpetrators of discrimination (60.0% vs 25.8%, *p* < 0.001), whereas this difference was not significant by gender (34.0% vs 24.7%, *p* = 0.178). Women and respondents born outside of Scandinavia were significantly more likely to report medical staff and clinical supervisors as perpetrators of discrimination (*p* = 0.004 and *p* = 0.001 respectively).

### Witnessing discrimination

43.6% (*n* = 108) had witnessed discrimination against others. The most common discrimination witnessed was based on ethnicity (*n* = 78), followed by gender (*n* = 72) and religion (*n* = 33) (Table 4). 53.9% (*n* = 133/247) were unaware whether their institution had a system to report discrimination, and 5.7% (*n* = 14/247) answered that their institution did not have such a system.

**Table 4:** Frequency of witnessed discrimination based on the seven discrimination grounds

	**N (%)**
**Discrimination towards peer witnessed**	
Yes	108 (43.7%)
Unsure	39 (15.8%)
No	99 (40.1%)
**Discrimination witnessed based on…**	
Sex	72 (29.2%)
Ethnicity	78 (31.6%)
Religion	33 (13.4%)
Sexuality	23 (9.3%)
Age	11 (4.5%)
Gender identity	9 (3.6%)
Disability	8 (3.2%)

### Thematic analysis

Free-text answers were sorted into seven convergent themes listed in the conceptual framework of “master suppression techniques”.

#### Making invisible

The master suppression technique of *“making invisible”* is defined as *“silencing or marginalizing people by ignoring them; being belittled or being communicated to as if you don’t matter or what you do or say is not important in the context”* [13].

Respondents who were women or of non-Caucasian ethnicity expressed feelings of invisibility where supervisors would preferentially offer opportunities, quiz or allocate more speaking time in group discussions to men or Caucasian students with increased tendencies to interrupt women when speaking. Some women also commented that they were frequently mistaken as nursing staff, whereas men were generally presumed to be medical students or even specialist physicians. Women also perceived more frequent expectations on them to do nursing tasks, such as making patients’ beds and serving food, whereas this was rarely asked of men. One respondent shared an example of a Caucasian respondent who experienced preferential treatment at the expense of a peer who was “made invisible”:

*“There are too many stories to share… It is sad to see especially the ethnic discrimination which is allowed to occur so directly and unashamedly. This can be not wanting to have eye contact with my colleague wearing a head scarf, and rather having eye contact with me, even though I am not involved in the patient care, or hearing staff speak about patients condescendingly, or being questioned when I stand up for patients of color. Sometimes I am ashamed when I put on my scrubs because I know that our health system does not practice the “anti-discrimination” in the way we say we do.”* - Woman, 30 years old, born in Sweden.

Conversely, multiple respondents described a recurring problem on obstetrics and gynaecology rotations, where men would have fewer opportunities to examine patients, and men of non-Caucasian descent, felt even more likely to be excluded, often with the presumption that patients would not consent.

#### Ridicule

*“Ridicule”* is defined as *“being mocked or laughed to scorn because of attributes relating to discrimination grounds”* [13]. *“Harmless jokes”* relating to various discrimination grounds were described by multiple respondents:

*“I am a Christian and have throughout the program experienced the presumption that we are all atheists. One lecturer asked the whole class if anyone had read the bible. I didn’t dare to raise my hand and no one else did either. When no one did, the lecturer said that it was good that no one had, because the Bible is just full of crap. I felt sad and as if I did not belong”. –* Woman, born in Scandinavia, bearing visible signs of a specific religion.

*“A supervisor on a clinical rotation where we were in the operating theatre and their colleague started “joking” about which anesthetic he would use if he would rape somebody. He then told me that as a student, if one is to work there you can’t be too sensitive. I felt like I was not about to report this since the supervisor had a strong position in the hospital and I was afraid it would impact me in the future”. –* Woman, 27 years old, born in Sweden.

#### Withholding information

“Withholding information” is defined as “withholding information or addressing important issues when certain individuals are not present including making decisions in informal places inaccessible to some people” [13]. This was described by multiple students of non-Caucasian descent who described that they had felt excluded from group projects with Caucasian peers, and by one respondent who had a physical disability which prevented her from accessing spaces where decisions were made:

*“I had a difficult condition requiring walking aids for one semester before I managed to get surgery….. and I couldn’t walk in stairs. Our university is not welcoming to people with limited mobility. I missed lectures and/or meetings because I couldn’t get to those places and other students didn’t want to find an alternative place that was accessible to me.” -* Woman, born outside of Europe.

#### Double punishment

*“Double punishment”* is defined as *““Damned if you do and damned if you don’t”, for example being blamed if you leave early to pick up children from daycare, but also considered a bad parent if you stay late at work”* [13]. Multiple respondents described the feeling of having to prove their competencies due to presumed prejudices, primarily associated with non-Caucasian ethnicity and being a woman, but also being questioned when pursuing their ambitions. This included a clinical supervisor telling women students that they should be less shy if they want more opportunities and supervisors discouraging women from pursuing time-intensive careers, particularly surgical specialties, due to difficulties in balancing it with family duties.

*“In medical school there is ethnic discrimination at every level… The discrimination is very subtle, but there are a lot of prejudices about those of us who don’t look Scandinavian. We constantly need to excel and can’t make mistakes if we want to be treated like our colleagues with Scandinavian descent. Many supervisors demand constant proof that we are competent to not treat us like incompetent idiots, whereas it is the opposite for our Scandinavian peers. Everyone comes late at some point. I have seen the same Scandinavian students come late almost every day and still be greeted with a positive attitude from supervisors and a brief recap of what they have missed, but I have seen the same supervisor be outraged when a colleague with a different background came late although he never comes late. So, on top of the program being demanding it itself, there are additional expectations on us with another ethnic background due to these prejudices.” –* Non-Caucasian man, born in Sweden.

*“I have always experienced that I need to be better than my peers, due to my ethnic background, to get the same opportunities. I have leadership positions in multiple organizations and taken on extra work but still see that peers with much fewer merits have gotten jobs where I wasn’t even invited for an interview. When I finally got an intern position, the employer wanted that I would send a proof of Swedish citizenship. I asked my classmates if they had ever had to do the same and no one had even heard of anything like this. I feel a lot of hopelessness in applying for internship/residency, due to many hospitals mainly recruiting by “local affiliation”. Since I have an immigration background, I do not have any “local affiliations”. –* Non-Caucasian woman, born in Sweden.

Respondents also shared *“compliments”* that they had received, perceived to contain underlying sentiments resembling micro-aggressions:

*“It has been everything from consultant physicians literally using the n-word in front of a black classmate to micro-aggressions like “wow, how have YOU come all the way here, how GOOD you are” (to a black, Muslim female peer student).” –* Non-Caucasian woman, born in Sweden.

#### Blame and shame

*“Blame and shame”* is defined as *“being held responsible for something that you are not”* [13]. One example of this was comments regarding the term *“cultural pain”*, an expression referring to exaggerated symptoms expressed by women of non-Caucasian descent. This expression was highlighted as one example of racism where the patient was held accountable for their mistreatment. Similarly, sociocultural barriers were described in situations pertaining to practical examination skills, where women expressed discomfort when expected to practice such skills on peers when wearing limited clothing. Questioning of students wearing head scarves was also described with undertones of *“blame and shame”.*

*“I often feel like my head scarf is questioned. ‘Is that your own? You are not allowed to wear it in the operating theatre’ or “you have to hide the head scarf entirely under your OR cap, nothing is allowed to show”, however, the same people never comment on other people’s hair falling outside the cap (which happens in 99% of cases in my experience). One time in the coffee room, one staff member started talking about pork and how much pork he/she ate. Of course, one is allowed to speak about what they eat, but it felt like it was aimed towards me (I am visibly Muslim), as if he/she thought it would have the same effect on me as garlic on vampires.” –* Non-Caucasian woman, born in Sweden.

#### Objectifying

*“Objectifying”* means *“treating a person as a commodity or an object.”* Women expressed feeling like they were not taken seriously, often called *“little girl”* or *“sweet girl”* by patients, with this leading to a feeling that they need to *“earn”* the respect from patients:

*“Some people don’t take young women as seriously as elder peers or men. I have also been called “pretty girl” at the hospital (when in my “doctor role”). I understood it was not meant in a mean way, but it didn’t feel good.”* – Caucasian woman, born in Sweden

#### Violence or threats of violence

Violence or threats of violence is defined by Nissen and Ås as *“Violence, including sexual violence, or threats of violence”* [13]. Comments of sexual nature or attempts of sexual harassment were described both from patients and senior clinical staff, with perceived limited ability to speak up against this due to power imbalances and fears of reprisals:

*“I was alone in a room with a male supervisor, 30 years older than me. He stood next to me, in front of the door and whispered: ‘Can I look a bit at you now?’ I experienced this situation as threatening. I told another male supervisor about this situation, and he said I should take it as a compliment.” –* Woman, born in Scandinavia

## Discussion

Sweden is considered one of the world’s most equitable countries – a welfare state with structural policies to reduce societal inequities, and legislation to prevent, report, and act upon discriminatory behaviour. Despite this, our study shows evidence that 41.7% of medical students in Sweden still experience discrimination, including mistreatment based on all seven discrimination grounds, although the prevalence of discrimination is lower than the levels reported in the systematic review by Fnais et al. of 51 studies of harassment and discrimination in six high- and middle-income countries (59.4%, 95% CI: 52.0%-66.7%) [3]. In our study, women and non-binary students, those perceived to be of a “non-Caucasian” ethnicity, and those born outside of Scandinavia were significantly more likely to have experienced discrimination. This pattern appears to be true also for lesbian, gay, and bisexual (LGB) students and students with visible signs of disability, although the small samples prevent any robust conclusions. An American study of over 20,000 medical students report similar susceptibilities of women, racial minorities, and LGB students to discrimination [18].

A third of the medical students in our study reported gender-based discrimination, which is similar to the findings of the Swedish Medical Association survey of recent medical graduates [19]. However, the majority who had experienced gender-based discrimination in our study-cohort reported low impact of this, whereas half of those experiencing religiously-based discrimination and over one-third of those experiencing ethnicity-based discrimination reported a large or extreme impact. Previous literature on mental health amongst medical students [20, 21] belonging to ethnic minorities suggests that there is a difference in impact of various types of discrimination. One alternative reason or contributing factor could be that women now constitute a majority of medical graduates in Sweden [9]. This can create a larger sense of belonging in the workforce, and more peers who can relate to the lived experiences as a woman in healthcare which may act as “protective factors” against heavy burden of discrimination.

In contrast, non-Caucasian ethnicities are still minorities amongst physicians in Sweden, potentiating the feeling of being an “imposter”, as fewer peers share the lived experiences that may be specific to underrepresented minorities. Moreover, the CHANGE study highlighted that for American medical students from “ethnic minorities”, ethnicity was a central component in their self-perception, which in turn was associated with poor well-being, when everyday discrimination was high [20]. Interestingly, racial discrimination was the third most common type of discrimination in our study and was reported by 42% of students who had experienced discrimination, with non-Caucasian students significantly more likely to have experienced discrimination. This can be compared to the previous systematic review by Fnais et al., which listed racial discrimination as the least common, with a prevalence of 26% [3]. However, racial discrimination was only studied in 7 of the 62 studies included by Fnais, so it is probable that racism and racial discrimination have been largely underreported. In recent years, multiple Swedish students of non-Caucasian ethnicity have raised their voices about perceived discrimination when applying for jobs post-graduation [22], and trainees and physicians have called for healthcare leadership to commit to anti-racism [23]. In our sample, we demonstrate that ethnic discrimination is especially frequent from patients and relatives; in line with recent investigations that have shown that many health services accommodate to patient requests to guarantee treatment by an “ethnically Swedish” doctor [24]. Similar findings have been reported previously with 23.5% of physicians in Sweden having experienced ethnically-based discrimination from patients and their relatives [25]. Yet, research regarding racism within healthcare has identified significant barriers to discourse and reporting; with ethnic discrimination still believed to be severely underreported [26, 27]. These barriers may be particularly pronounced in Sweden due to the lack of agreed-upon terminology and ethnicity-related data not regularly being collected, preventing health inequities from being tracked along with other welfare data [26]. This has been argued to be a result of notions of “Swedish exceptionalism” with the widespread self-perception of Swedish society as anti-racist, gender-equal and untainted by colonial legacy, further hindering progressive discourse and critical introspective analysis [26].

Discrimination does not occur in a vacuum – it takes place in the context of power imbalances and inequitable systems. In our study, the most common perpetrators of discrimination were clinical staff, followed closely by patients and their relatives. Medical students are particularly vulnerable as they are often in positions of dependence to receive high marks, credentials for future career opportunities, and acceptance in the team to ensure comfortable workplace relations [28], often limiting opportunities to speak up against injustices. Previous literature across different national contexts has similarly reported low levels of reporting [29, 30], with explanations of reporting seen as ineffective and fears of reprisals having been shown to be well-founded [29]. However, lack of reporting appears to also be due to limited awareness, as less than half of respondents were cognizant of existing reporting systems for discrimination, even though all seven medical faculties in Sweden have such systems in place.

As the first study to assess discrimination based on Swedish and EU legislation including seven intersecting discrimination grounds, we believe similar data is needed in other contexts to allow for multidimensional analysis, and consequentially, holistic solutions to address the problem. This should include institutional measures including raising awareness of existing reporting systems and assessing barriers to reporting. Previous literature has highlighted the important influence of the “hidden curriculum”, including attitudes, behaviors and values from students and teachers that perpetuate discriminatory cultures despite efforts to address these issues in the formal curriculum [31]. However, the breadth of this issue, including evidence of frequent discrimination from patients and relatives, also indicates the need for systemic change, arguably beyond the reach of medical educators. Finally, ignorance of an inequitable system is likely to perpetuate injustices further; hence, discrimination and the historical contexts in our curriculums should be expanded, and medical schools should take responsibility to safeguard its future workforce by lobbying against discriminatory movements including requests for “ethically Swedish” physicians.

## Limitations

This study is likely to evoke responder bias, where those with experiences of discrimination and/or interest in social justice, may be more likely to participate in this study. According to the Swedish National Board of Health and Welfare (Socialstyrelsen), 56% of medical interns (the first formal position after graduating medical school) in 2018 were women. In our sample, 59.5% of respondents were women, which reflects the proportion of women among the Swedish medical students. Unfortunately, the National Board of Health and Welfare does not have further demographic data on medical trainees and staff, which hinders full comparison between our sample and the target population, however based on the available demographic data, our sample appears to be representative of the target population. This study could also have some recall bias; however, we have tried to mitigate both these sources of biases by formulating a neutral study invitation and open questions. The study has a low response rate, which led the study to be underpowered to detect significant differences between the smaller sub-groups (for example those with visible signs of disability, *n* = 7), and makes the regression results for these smaller sub-groups hard to interpret. However, low response rates is a well-known problem for web-based surveys amongst medical students, possibly due to survey fatigue in addition to distribution challenges [32]. Hence, the findings should be seen as an indication of the scope of discrimination which warrants further research. We did not conduct any formal measurements of the related construct of workplace bullying, as this was neither the aim nor within the scope of our study. Finally, this study was not designed to address the intersectionality of discrimination, although the interconnected nature of social categorizations such as age, ethnicity and gender may be considered as overlapping and interdependent systems of discrimination. Yet, this study is an initial step to understanding the paradigms of discrimination in a “gender-equal” country, and the results should be followed up in a complementary study addressing the intersecting nature of these discrimination grounds.

## Conclusion

Medical students are frequent targets of discrimination, even in one of the world’s “most equal countries”. Discrimination occurs on multiple grounds with gender- and ethnicity-based discrimination the most common, and ethnicity- and religion-based discrimination appearing to have the largest impact. Future studies should seek to evaluate experiences based on the intersecting nature of discrimination grounds, adapted for local contexts and legislations.

## Supplementary Information

**Additional file 1: Table 1.** Survey. N/A=Not Applicable; *Scandinavia=Denmark, Norway, Sweden, Finland, Iceland, the Åland islands, and the Faroe Islands. **Figure 1.** Advertisement flyer used for survey dissemination. **Table 2.** Master suppression techniques as defined by Nissen and Ås, translated to English by the study authors.

## Data Availability

Raw data is not publicly available to maintain the confidentiality of survey respondents and avoid identification of individuals. For specific questions regarding the raw data, the corresponding author can be contacted.
