# Supplementary material for: Discrimination in an “equal country”—a survey amongst Swedish final-year medical students
Source: BMC Med Educ. 2022 Jun 27;22:503. doi: 10.1186/s12909-022-03558-6 (PMC9238226; doi:10.1186/s12909-022-03558-6)
Supplement: Supplementary file 1 — Additional file 1: Table 1. Survey. N/A=Not Applicable; *Scandinavia=Denmark, Norway, Sweden, Finland, Iceland, the Åland islands, and the Faroe Islands. Figure 1. Advertisement flyer used for survey dissemination. Table 2. Master suppression techniques as defined by Nissen and Ås, translated to English by the study authors. [file 12909_2022_3558_MOESM1_ESM.docx]

**Supplementary materials**

**Table 1.** Survey. N/A=Not Applicable; *Scandinavia=Denmark, Norway, Sweden, Finland, Iceland, the Åland islands, and the Faroe Islands.

| **Question number** | **Question** | **Answer format** | **Answer alternative** |
| --- | --- | --- | --- |
| 1 | Do you consent to participate? | Single answer | - Yes |
| 2 | Which semester in medical school are you in? (only students in the 10^th^ and 11^th^ semester can take part) | Single answer, categorical | - 10^th^ semester - 11^th^ semester |
| 3 | How old are you? (write the number only) | Numeric (free-text) | N/A |
| 4 | What gender do you identify as? | Single answer, categorical | - Female - Male - Non-binary - Don’t want to state |
| 5 | Where were you born? | Single answer, categorical | - In Sweden - In Scandinavia* (not Sweden) - In Europe (not Scandinavia) - Outside Europe |
| 6 | Do you think others perceive you as ethnically Swedish? | Single answer, categorical | - Yes - No - Don’t know - Don’t want to state |
| 7 | How would you describe your sexuality? | Single answer, categorical | - Bisexual - Heterosexual - Homosexual - Pansexual - Asexual - Intersexual - Other - Don’t want to state |
| 8 | Do you have any visible disability? | Single answer, categorical | - Yes - No - Don’t want to state |
| 9 | Do you have any visible signs which may lead others to think that you have any specific religious belief? | Single answer, categorical | - Yes - No - Don’t know   Don’t want to state |
| 10 | Do you feel that you have experienced discrimination during your time in medical school? | Single answer, categorical | - Yes - No - Don’t want to state |
| 11a | If 10=Yes; To what extent have you been affected by discrimination based on your sex? | Single answer, categorical | - Not at all - Small extent - Some extent - Large extent - Extreme extent |
| 11b | If 10=Yes; To what extent have you been affected by discrimination based on your non-binary gender identity or expression? | Single answer, categorical | - Not at all - Small extent - Some extent - Large extent - Extreme extent |
| 11c | If 10=Yes; To what extent have you been affected by discrimination based on your ethnicity? | Single answer, categorical | - Not at all - Small extent - Some extent - Large extent - Extreme extent |
| 11d | If 10=Yes; To what extent have you been affected by discrimination based on your religion or other belief? | Single answer, categorical | - Not at all - Small extent - Some extent - Large extent - Extreme extent |
| 11e | If 10=Yes; To what extent have you been affected by discrimination based on your disability? | Single answer, categorical | - Not at all - Small extent - Some extent - Large extent - Extreme extent |
| 11f | If 10=Yes; To what extent have you been affected by discrimination based on your sexuality? | Single answer, categorical | - Not at all - Small extent - Some extent - Large extent - Extreme extent |
| 11g | If 10=Yes; To what extent have you been affected by discrimination based on your age? | Single answer, categorical | - Not at all - Small extent - Some extent - Large extent - Extreme extent |
| 12a | If 10=Yes; Who discriminated against you? (mark all that apply) | Multiple answer, categorical | - Other medical students - Preclinical teachers - Medical staff / supervisors during clinical placements - Non-medical staff during clinical placements - Patients / patients’ relatives - Others (please specify) |
| 13a | If 10=Yes; How has the discrimination impacted your performance during medical school? | Single answer, categorical | - Not at all - Small extent - Some extent - Large extent - Extreme extent |
| 13b | If 10=Yes; How has the discrimination impacted your personal wellbeing? | Single answer, categorical | - Not at all - Small extent - Some extent - Large extent - Extreme extent |
| 13c | If 10=Yes; How has the discrimination impacted your self-esteem? | Single answer, categorical | - Not at all - Small extent - Some extent - Large extent - Extreme extent |
| 13d | If 10=Yes; How has the discrimination impacted your sense of personal security? | Single answer, categorical | - Not at all - Small extent - Some extent - Large extent - Extreme extent |
| 13e | If 10=Yes; How has the discrimination impacted your future career choices? | Single answer, categorical | - Not at all - Small extent - Some extent - Large extent - Extreme extent |
| 14a | Have you witnessed discrimination towards someone else during medical school? | Single answer, categorical | - Yes - No - Don’t know - Don’t want to state |
| 14b | If 14a=Yes; For which discrimination grounds have you witnessed discrimination towards someone else? | Multiple answer, categorical | - Sex - Non-binary gender identity or expression - Ethnicity - Religion - Disability - Sexuality - Age - Other - Don’t want to state |
| 15 | Does your institution have a formal reporting system for discrimination? | Single answer, categorical | - Yes - No - Don’t know |
| 16 | This is the final question. Please use this space to share your personal experiences or examples of discrimination that you have experienced or witnessed during medical school. How did these events affect you, other healthcare staff or patients? Please refrain from sharing information which can be identifiable (for example name, exact title, university/hospital name etc). | Free-text | N/A |


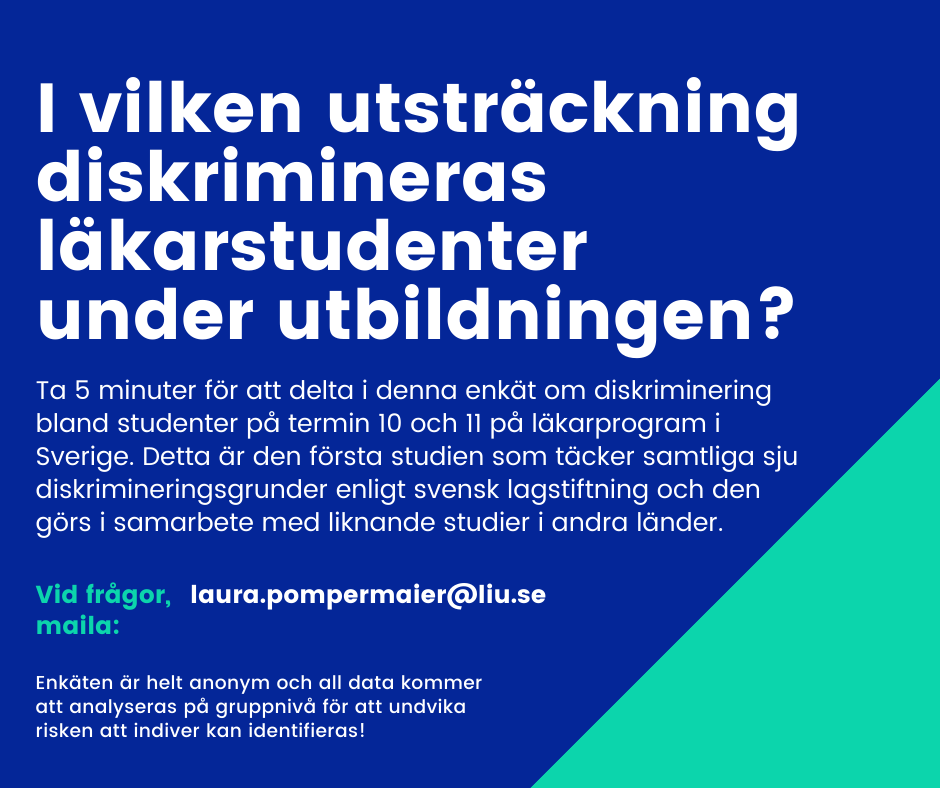


**Figure 1.** Advertisement flyer used for survey dissemination.

Translation: To what extent do medical students experience discrimination during their education? Take 5 minutes to take part in this survey about discrimination amongst final-year medical students in Sweden. This is the first study to cover all seven discrimination grounds according to Swedish law and it is conducted in collaboration with similar studies in other countries. If you have questions, contact: [laura.pompermaier@liu.se](mailto:laura.pompermaier@liu.se). The survey is anonymous, and all data will be analyzed at aggregated level to avoid risk of personal identification.

**Table 2.** Master suppression techniques as defined by Nissen and Ås, translated to English by the study authors.

| **Master suppression technique** | **Definition** |
| --- | --- |
| Making invisible | Silencing or marginalizing people by ignoring them; being belittled or being communicated to as if you don’t matter or what you do or say is not important in the context. |
| Ridicule | Being mocked or laughed to scorn because of attributes relating to discrimination grounds. |
| Withholding information | Withholding information or addressing important issues when certain individuals are not present including making decisions in informal places inaccessible to some people. |
| Double punishment | “Damned if you do and damned if you don’t”, for example being blamed if you leave early to pick up children from day care, but also considered a bad parent if you stay late at work. |
| Blame and shame | Being held responsible for something that you are not. |
| Objectifying | Treating a person as a commodity or an object. |
| Violence or threats of violence | Violence, including sexual violence, or threats of violence. |
